# *In silico* clustering of *Salmonella* global gene expression data reveals novel genes co-regulated with the SPI-1 virulence genes through HilD

**DOI:** 10.1038/srep37858

**Published:** 2016-11-25

**Authors:** Irma Martínez-Flores, Deyanira Pérez-Morales, Mishael Sánchez-Pérez, Claudia C. Paredes, Julio Collado-Vides, Heladia Salgado, Víctor H. Bustamante

**Affiliations:** 1 Programa de Genómica Computacional, Centro de Ciencias Genómicas, Universidad Nacional Autónoma de México, Cuernavaca, Morelos 62210, México; 2 Departamento de Microbiología Molecular, Instituto de Biotecnología, Universidad Nacional Autónoma de México, Cuernavaca, Morelos 62210, México

## Abstract

A wide variety of *Salmonella enterica* serovars cause intestinal and systemic infections to humans and animals. *Salmonella* Patogenicity Island 1 (SPI-1) is a chromosomal region containing 39 genes that have crucial virulence roles. The AraC-like transcriptional regulator HilD, encoded in SPI-1, positively controls the expression of the SPI-1 genes, as well as of several other virulence genes located outside SPI-1. In this study, we applied a clustering method to the global gene expression data of *S. enterica* serovar Typhimurium from the COLOMBOS database; thus genes that show an expression pattern similar to that of SPI-1 genes were selected. This analysis revealed nine novel genes that are co-expressed with SPI-1, which are located in different chromosomal regions. Expression analyses and protein-DNA interaction assays showed regulation by HilD for six of these genes: *gtgE*, *phoH*, *sinR, SL1263 (lpxR*) and *SL4247* were regulated directly, whereas *SL1896* was regulated indirectly. Interestingly, *phoH* is an ancestral gene conserved in most of bacteria, whereas the other genes show characteristics of genes acquired by *Salmonella*. A role in virulence has been previously demonstrated for *gtgE*, *lpxR* and *sinR*. Our results further expand the regulon of HilD and thus identify novel possible *Salmonella* virulence genes.

The genus *Salmonella* groups Gram-negative bacteria that can infect humans and a great variety of animals, causing self-limiting enteritis or a systemic disease^1^,^2^. *Salmonella* comprises only two species, *bongori* and *enterica*, the latter is further divided into six subspecies and around 2500 serotypes or serovars^3^. *S. enterica* serovar Typhimurium (*S*. Typhimurium) can cause intestinal or systemic infections in different hosts; thus, it is widely used as a model to study the molecular virulence mechanisms of *Salmonella*^4^.

Around one-third of the *Salmonella* genome has been shaped by horizontal events; most of the acquired genes are clustered in regions denominated islands^5^,^6^. *Salmonella* pathogenicity island 1 (SPI-1) is a chromosomal region conserved in the two *Salmonella* species, which contains 39 genes that code for a type 3 secretion system (T3SS-1), different effector proteins and their chaperones, as well as for transcriptional regulators that control the expression of the genes within this island^4^,^7^. The T3SS and effector proteins encoded in SPI-1 are required for *Salmonella* invasion into intestinal epithelial cells and thus for the intestinal colonization leading to enteritis^1^,^4^,^8^. *In vivo,* the SPI-1 genes are expressed when *Salmonella* is in the intestinal lumen, associated with the epithelium or with extruding enterocytes^9^, and also in a subpopulation of *Salmonella* hyperreplicating in the cytosol of epithelial cells^10^. *In vitro,* the SPI-1 genes are expressed during the early stationary phase when *Salmonella* is grown in nutrient-rich media, such as the Luria-Bertani (LB) medium, and their expression is regulated by growth-phase, temperature, osmolarity, oxygen tension, long- and short-chain fatty acids concentration, pH, iron level and bile^11^,^12^,^13^,^14^,^15^,^16^,^17^,^18^,^19^.

Expression of the SPI-1 genes is controlled through a regulatory cascade formed by the transcriptional regulators HilD, HilA and InvF, encoded within this island^4^,^20^. HilD, a member of the AraC/XylS family of transcriptional regulators, directly induces the expression of HilA^21^,^22^,^23^, a regulator with an OmpR/ToxR-like DNA binding domain, which in turn, activates the expression of InvF^24^,^25^, another AraC/XylS-like regulator; HilA and InvF activate the expression of the SPI-1 genes encoding the T3SS-1 components and effector proteins, respectively^4^,^20^. Although HilD has a dominant role, the expression of HilA, and thus the SPI-1 genes, depends on a complex feed-forward positive regulatory loop formed by HilD, HilC and RtsA^23^,^26^; both HilC and RtsA are AraC-like regulators and bind the DNA sequence recognized by HilD^27^,^28^. HilD also induces directly, or indirectly through HilA, InvF or other regulators, the expression of several other virulence genes, including genes located in SPI-2, SPI-4, SPI-5 and other islands^4^,^14^,^29^,^30^,^31^,^32^,^33^,^34^. Furthermore, HilD directly controls the expression of the *flhDC* operon encoding FlhDC, the master transcriptional complex required for the expression of flagellar and chemotaxis genes^30^,^35^. Interestingly, FlhDC induces the expression of FliZ that somehow increases activity of HilD and thus the expression of the SPI-1 genes^36^. Therefore, HilD and FlhDC form a positive regulatory loop that co-regulates the expression of the SPI-1 and flagellar/chemotaxis genes. Important to note, like the SPI-1 genes, the flagellar/chemotaxis genes are also necessary for the invasion of *Salmonella* into host cells^4^,^37^,^38^. Thus, the HilD regulon comprises genes encoding different cellular functions that make to *Salmonella* an intestinal pathogen.

Gene expression is coordinated to carry out the different cellular activities; therefore, co-expression analyses can be used to infer biological functions of uncharacterized genes, as well as to identify the regulatory pathways governing the co-expression relationships. The high amount of global gene expression data that is currently available offers opportunities to investigate the gene co-expression networks in many organisms. COLOMBOS is a database that integrates a collection of transcriptomics results from both microarray and RNA-seq experiments for several prokaryotic species, including *Salmonella*^39^.

In this work, we identified genes co-expressed with the SPI-1 genes, by applying a clustering method to the *S*. Typhimurium SL1344 global gene expression data from the COLOMBOS database. This analysis indicates that most genes known to be directly or indirectly regulated by HilD are indeed co-expressed with the SPI-1 genes, including acquired genes located outside SPI-1 and the flagellar/chemotaxis ancestral genes. Interestingly, our results revealed nine novel genes that are co-expressed with SPI-1: *gtgE*, *phoH*, *sinR*, *SL1344_1028 (SL1028*), *SL1344_1263 (SL1263* or *lpxR*), *SL1344_1896 (SL1896*), *SL1344_3812 (SL3812*), *SL1344_4247 (SL4247*) and *SL1344_4433 (SL4433*). Expression analyses and protein-DNA interaction assays show that HilD regulates six of these genes: *gtgE*, *phoH*, *sinR, lpxR* and *SL4247* were regulated directly, whereas *SL1896* was regulated indirectly. Sequence and genome context analyses indicate that *gtgE*, *sinR*, *lpxR*, *SL1896* and *SL4247* are genes that were acquired by *Salmonella*, whereas *phoH* is an ancestral gene. A role in virulence has been previously determined for *gtgE*, encoding an effector protein secreted by the two T3SSs of *Salmonella*; *lpxR*, that codes for an outer membrane protein that removes the 3´-acyloxyacyl group of lipid A; and *sinR*, encoding a putative LysR-family transcriptional regulator. The *phoH* gene encodes a protein that has an ATP-binding activity, which is conserved in most of bacteria, whereas the *SL4247* and *SL1896* genes encode hypothetical proteins. Therefore, our results further expand the virulence regulon of HilD and identify novel genes possibly involved in the pathogenesis of *Salmonella*.

## Results

### Identification of genes co-expressed with SPI-1

**Figure 1:**
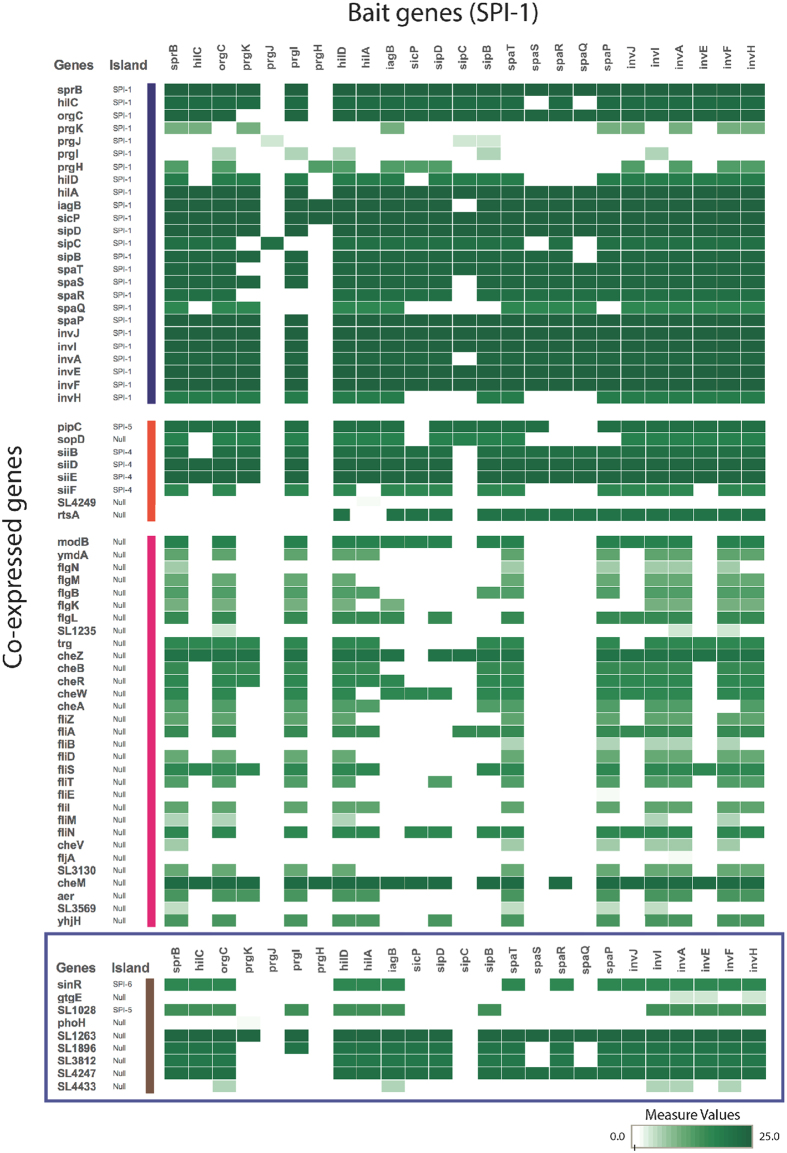
Genes co-expressed with SPI-1. The heat map represents the frequency with which each gene, shown at the left side of this Fig. (co-expressed genes), was clustered with the corresponding SPI-1 gene used as the bait, shown above this Fig. The intensity of the green color in the heat map indicates the score obtained for each co-expressed gene, based in the color bar shown below the figure, ranging from Measure Values of 0 to 25. The frequency scores for all the co-expressed genes are displayed in Table S1 in Supplementary File 1. The co-expressed genes are classified in four groups: genes located in SPI-1, genes known to be co-regulated with SPI-1 that are located in other genomic islands, flagellar/chemotaxis genes and novel genes that are co-expressed with SPI-1, which are indicated with dark blue, orange, pink and brown color bars, respectively. For a better visualization, the novel genes that are co-expressed with SPI-1 are boxed. The left side of this Fig. also show whether the genes co-expressed with SPI-1 are located in any SPI. Null, indicates not located in any SPI.

To identify genes co-expressed with SPI-1, we applied a clustering method to the genome-wide expression data of *S*. Typhimurium SL1344 from COLOMBOS, which generated a variety of clusters containing genes with a similar expression pattern. Then, SPI-1 genes were used independently as a bait to select those clusters that should include genes expected to be co-expressed with SPI-1. This analysis generated scores indicating the frequency with which a gene is clustered with the corresponding SPI-1 gene used as the bait (Table S1 in Supplementary File 1). For a better visualization, these data are represented in a heat map (Fig. 1). The co-expression pattern for each SPI-1 gene used as the bait show some differences; there are genes that were clustered with some SPI-1 genes but not with others (Fig. 1), which could be due to subtle differences in regulation between the SPI-1 genes or variations in the results from the global expression experiments analyzed. Therefore, the use as the bait of several SPI-1 genes increased the possibility to find genes co-expressed with SPI-1.

Taken together, the results from our clustering analysis show that many genes known to be regulated by HilD, directly or through HilA, InvF or FlhDC, including the SPI-1 genes themselves and genes located in different genomic islands, as well as flagellar/chemotaxis genes^4^,^34^,^40^,^41^, are indeed co-expressed with SPI-1 (Fig. 1; Table S1 in Supplementary File 1). Interestingly, the *gtgE*, *phoH*, *sinR*, *lpxR, SL1028*, *SL1896*, *SL3812*, *SL4247* and *SL4433* genes were also found to be co-expressed with SPI-1 (Fig. 1; Table S1 in Supplementary File 1). Another clustering analysis, now using these nine genes as the bait, showed groups of co-expressed genes very similar to those obtained by using the SPI-1 genes as the bait (Fig. S1 in Supplementary File 2 and Table S2 in Supplementary File 1), further supporting the link in expression of all these genes.

Thus, these results indicate that the clustering method that we used was successful to find genes co-expressed with SPI-1, identifying *gtgE*, *phoH*, *sinR*, *lpxR, SL1028*, *SL1896*, *SL3812*, *SL4247* and *SL4433* as novel genes co-expressed with SPI-1.

### HilD, but not HilA or InvF, regulates the expression of *gtgE*, *phoH*, *sinR*, *lpxR*, *SL1896* and *SL4247*

**Figure 2:**
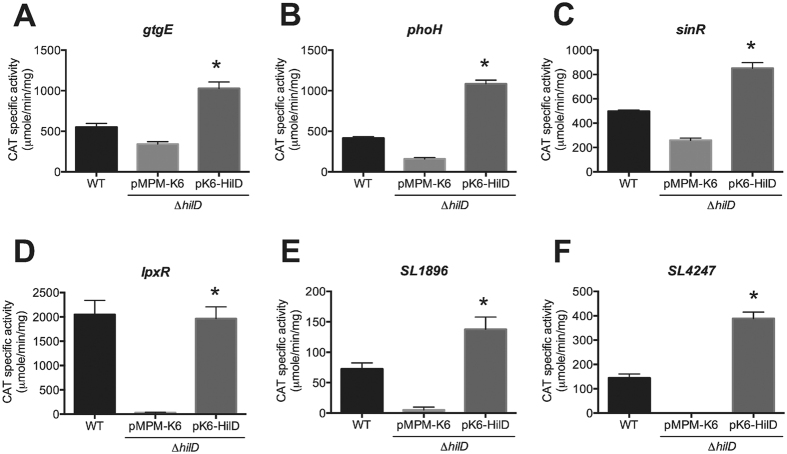
HilD positively regulates *gtgE*, *phoH*, *sinR*, *lpxR*, *SL1896* and *SL4247* in *S*. Typhimurium. Expression of the *gtgE-cat* (**A**), *phoH-cat* (**B**), *sinR-cat* (**C**), *lpxR-cat* (**D**), *SL1896-cat* (**E**) and *SL4247-cat* (**F**) transcriptional fusions contained in plasmids pgtgE-cat, pphoH-cat, psinR-cat, plpxR-cat, pSL1896-cat and pSL4247-cat, respectively, was tested in the WT *S*. Typhimurium strain SL1344 and its isogenic Δ*hilD* mutant carrying the vector pMPM-K6 or the plasmid pK6-HilD, which expresses HilD under an arabinose-inducible promoter. CAT-specific activity was determined from samples collected of bacterial cultures grown for 9 h in LB medium at 37 °C. Expression of HilD from pK6-HilD was induced by adding 0.001% L-arabinose to the medium at the beginning of the bacterial cultures. The data are the average of three independent experiments performed in duplicate. Bars represent the standard deviations. *Expression statistically different with respect to that shown by the same transcriptional fusions in the Δ*hilD* mutant carrying the vector pMPM-K6, *P*-value < 0.001.

To determine whether the expression of the novel genes found to be co-expressed with the SPI-1 genes is controlled by the major transcriptional regulators encoded in SPI-1, HilD, HilA and InvF, transcriptional fusions of these genes to the *cat* gene were constructed in plasmid pKK232–8, an expression reporter system that we have successfully used in *S*. Typhimurium^14^,^42^,^43^,^44^. These transcriptional fusions carry the intergenic region upstream of the respective gene tested. The chloramphenicol acetyl transferase (CAT)-specific activity directed by plasmids carrying the transcriptional fusions *gtgE-cat*, *phoH-cat*, *sinR-cat*, *lpxR-cat, SL1028-cat*, *SL1896-cat*, *SL3812-cat*, *SL4247-cat* or *SL4433-cat*, was determined in wild type (WT) *S*. Typhimurium strain SL1344 and its isogenic Δ*hilD*, Δ*hilA* and Δ*invF* mutants, grown in LB medium a 37 °C, conditions that favor the expression of the SPI-1 genes^14^,^42^,^44^. As a control, the expression of a *cat* transcriptional fusion of *invF*, which is positively regulated by HilD through HilA, was also assessed. Expression of the *gtgE-cat*, *phoH-cat*, *sinR-cat*, *lpxR-cat*, *SL1896-cat* and *SL4247-cat* fusions was decreased in the Δ*hilD* mutant, but not in the Δ*hilA* and Δ*invF* mutants, with respect to their expression levels shown in the WT strain (Table 1). Furthermore, the plasmid pK6-HilD, expressing HilD from an arabinose-inducible promoter, was able to increase the expression of these fusions in the Δ*hilD* mutant to WT levels or even higher (Fig. 2A–F). In contrast, the expression of the *SL1028-cat, SL3812-cat* and *SL4433-cat* fusions was not significantly reduced in the Δ*hilD*, Δ*hilA* or Δ*invF* mutants and, as expected, the expression of the *invF-cat* fusion was decreased in the Δ*hilD* and Δ*hilA* mutants, but not in the Δ*invF* mutant (Table 1). The *SL1028-cat* fusion showed a very low level of CAT activity (Table 1), which is consistent with the very low level of expression previously detected for *SL1028* by RNA-seq-based transcriptomic analyses^17^.

These results show that HilD positively controls the expression of the *gtgE*, *phoH*, *sinR*, *lpxR*, *SL1896* and *SL4247* genes, independently of HilA and InvF.

### HilD induces the expression of *gtgE*, *phoH*, *sinR*, *lpxR* and *SL4247*, but not that of *SL1896*, in the absence of other *Salmonella*-specific regulators

**Figure 3:**
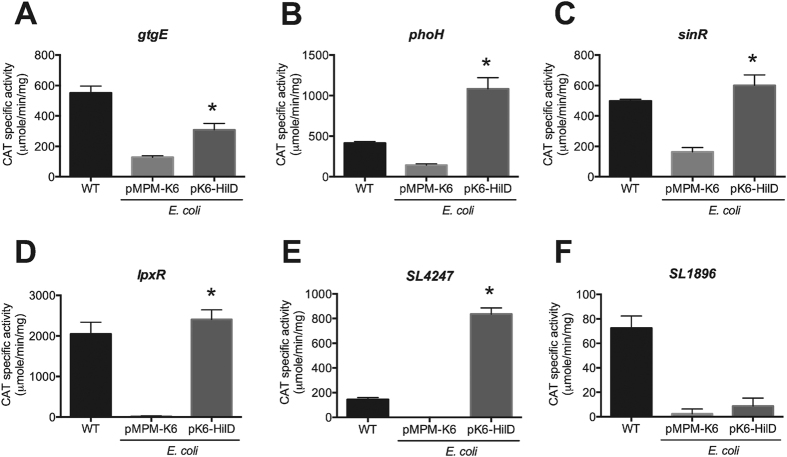
HilD induces the expression of *gtgE*, *phoH*, *sinR*, *lpxR* and *SL4247*, but not *SL1896*, in *E. coli* MC4100. Expression of the *gtgE-cat* (**A**), *phoH-cat* (**B**), *sinR-cat* (**C**), *lpxR-cat* (**D**), *SL4247-cat* (**E**) and *SL1896-cat* (**F**) transcriptional fusions contained in plasmids pgtgE-cat, pphoH-cat, psinR-cat, plpxR-cat, pSL4247-cat and pSL1896-cat, respectively, was tested in the *E. coli* MC4100 strain carrying the vector pMPM-K6 or the plasmid pK6-HilD, which expresses HilD under an arabinose-inducible promoter. CAT-specific activity was determined from samples collected of bacterial cultures grown for 9 h in LB medium at 37 °C. Expression of HilD from pK6-HilD was induced by adding 0.001% L-arabinose to the medium at the beginning of the bacterial cultures. The data are the average of three independent experiments performed in duplicate. Bars represent the standard deviations. *Expression statistically different with respect to that shown by the same transcriptional fusions in the presence of the vector pMPM-K6, *P*-value < 0.001.

*E. coli* K-12 lacks around 1,400 genes present in *S*. Typhimurium, including several encoding transcriptional regulators, such as HilD, located in the SPIs and other *Salmonella* islands. Therefore, to determine whether the HilD-mediated expression of the *gtgE*, *phoH*, *sinR*, *lpxR*, *SL1896* and *SL4247* genes requires additional *Salmonella*-specific regulators, we determined the expression of the transcriptional fusions of these genes in the *E. coli* MC4100 strain carrying the plasmid pK6-HilD or the vector pMPM-K6Ω. As controls, the expression of *cat* transcriptional fusions of the *hilA* and *ssaG* genes, which are controlled by HilD directly and indirectly, respectively, were also assessed. It is important to note that the *E. coli* MC4100 strain carries a frameshift mutation in the *flhDC* operon and thus it does not express the flagellar transcriptional regulator FlhDC^45^,^46^; HilD positively regulates the expression of FlhDC in *S*. Typhimurium^30^,^35^. The expression of all these fusions tested was reduced in the *E. coli* MC4100 strain carrying the vector pMPM-K6Ω, with respect to their respective expression level shown in the WT *S*. Typhimurium strain (Fig. 3A–F), which is consistent with their positive regulation by HilD. The expression of HilD from plasmid pK6-HilD increased the activity of the fusions *gtgE-cat*, *phoH-cat*, *sinR-cat*, *lpxR-cat*, and *SL4247-cat*, similar at their respective levels reached in the WT *S*. Typhimurium strain or even higher (Fig. 3A–E), showing that the HilD-mediated expression of *gtgE*, *phoH*, *sinR*, *lpxR* and *SL4247* does not need any other *Salmonella*-regulator nor FlhDC. In contrast, the expression of HilD from plasmid pK6-HilD did not increase the activity of the *SL1896-cat* fusion (Fig. 3F), indicating that HilD induces the expression of *SL1896* through a factor not present in *E. coli* MC4100. As expected, the presence of HilD also induced the activity of the *hilA-cat* fusion, but not that of the *ssaG-cat* fusion (data not shown). To investigate whether HilD induces the expression of *SL1896* through FlhDC, a Δ*flhDC* mutant derivative of *S*. Typhimurium SL1344 was constructed and the activity of the *SL1896-cat* fusion was determined in this strain. Additionally, a *cat* transcriptional fusion of *trg*, a gene regulated by FlhDC^41^,^47^, was constructed and analyzed as a control in these assays. The *SL1896-cat* fusion showed similar expression levels in the WT and Δ*flhDC* mutant strains (72 ± 9 and 82 ± 7, respectively), whereas the activity of the *trg-cat* fusion was drastically decreased in the Δ*flhDC* mutant (19 ± 8), with respect to its expression levels shown in the WT strain (154 ± 6), indicating that FlhDC is not required for the expression of *SL1896* in the growth conditions tested.

Thus, these data strongly support that HilD directly controls the expression of the *gtgE*, *phoH*, *sinR*, *lpxR* and *SL4247* genes, and indirectly, through a regulator found in *S*. Typhimurium but not in *E. coli* MC4100, that of the *SL1896* gene.

### HilD binds to the regulatory regions of *gtgE*, *phoH*, *sinR*, *lpxR* and *SL4247*, but not to that of *SL1896*

**Figure 4:**
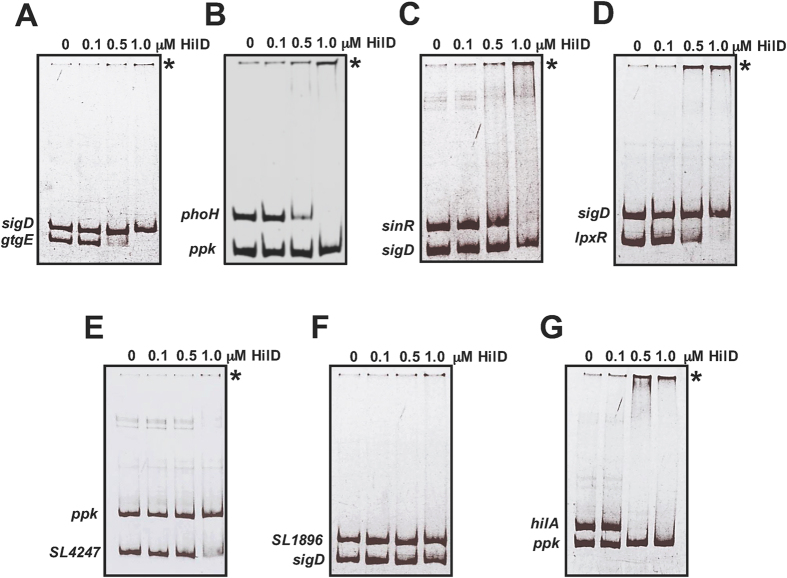
HilD binds to the regulatory region of *gtgE*, *phoH*, *sinR*, *lpxR* and *SL4247*, but not to that of *SL1896*. MBP-HilD binding to the DNA fragments contained in the *gtgE-cat* (**A**), *phoH-cat* (**B**), *sinR-cat* (**C**), *lpxR-cat* (**D**), *SL4247-cat* (**E**) and *SL1896-cat* (**F**) transcriptional fusions was analyzed by competitive nonradioactive EMSAs. As a positive control, the regulatory region of *hilA* was also assessed (**G**), and as a negative internal control, a DNA fragment containing the regulatory region of *ppk* or *sigD* was included in each DNA-binding reaction. PCR-amplified and purified DNA fragments were incubated with increasing concentrations (0 to 1 μM) of purified MBP-HilD fusion protein. The DNA-protein complexes (indicated by an asterisk) were resolved in a nondenaturing 5% polyacrylamide gel and stained with ethidium bromide.

To further define whether the HilD-mediated regulation of *gtgE*, *phoH*, *sinR*, *lpxR*, *SL1896* and *SL4247* is direct or indirect, we analyzed the interaction of HilD with the regulatory region of these genes. Affinity-purified maltose-binding protein (MBP)-HilD, which is active *in vivo* and specifically binds to HilD-target genes *in vitro*^14^,^29^, and the DNA fragments contained in the respective transcriptional fusion of each gene, were used to perform electrophoretic mobility shift assays (EMSAs). As a positive control, a DNA fragment containing the regulatory region of *hilA* was also assessed. Additionally, a DNA fragment containing the intergenic region upstream of *ppk,* a gene not regulated by HilD, or *sigD,* a gene not directly regulated by HilD, was included in the binding reactions as an internal negative control. MBP-HilD specifically bound the DNA fragments of *gtgE*, *phoH*, *sinR, lpxR*, *SL4247* and, as expected, that of *hilA,* at concentrations of 0.1 to 1.0 μM; in contrast, at the same concentrations it did not bind the DNA fragment of *SL1896*, or those of the negative controls, *ppk* and *sigD* (Fig. 4A–G). At concentrations higher than 1.5 μM, MBP-HilD bound most of the DNA fragments tested, including the negative controls, indicating that it binds non-specifically at these concentrations (data not shown). In order to identify putative HilD-binding sites, we scanned the regulatory regions of the *gtgE*, *phoH*, *sinR*, *lpxR* and *SL4247* genes with PSSMs representing the two HilD-binding consensus sequences reported previously^27^,^30^. Some HilD-binding sites were predicted in these genes (Fig. S2 in Supplementary File 2), which is consistent with their direct regulation by HilD.

Together with the expression analyses, these binding assays demonstrate that HilD directly regulates the expression of the *gtgE*, *phoH*, *sinR*, *lpxR* and *SL4247* genes, and indirectly that of the *SL1896* gene.

## Discussion

Co-regulation can ensure the coordinated expression of genes located in different chromosomal regions whose products are required for specific cellular functions. For instance, the transcriptional regulator HilD, encoded in SPI-1, positively controls the expression of the genes within this island, as well as several other genes located outside SPI-1, which mediate *Salmonella* invasion of host cells^4^,^30^. Additionally, HilD also positively regulates several genes necessary for *Salmonella* replication inside host cells, including the SPI-2 genes^4^,^14^,^29^.

In this work, by applying a clustering method to *S*. Typhimurium SL1344 global expression data from the COLOMBOS database, we show that most of the known genes regulated by HilD, including the flagellar/chemotaxis genes, are indeed co-expressed with SPI-1; moreover, nine novel genes that are co-expressed with SPI-1 were identified: *gtgE*, *phoH*, *sinR*, *SL1028*, *lpxR*, *SL1896*, *SL3812*, *SL4247* and *SL4433*. Furthermore, we demonstrate that HilD is required for the expression of the *gtgE*, *phoH*, *sinR*, *lpxR*, *SL1896* and *SL4247* genes, but not for the *SL1028*, *SL3812* and *SL4433* genes, when *S*. Typhimurium SL1344 is grown in conditions that favor the expression of the SPI-1 genes. FlhDC, the master regulator of the flagellar/chemotaxis genes, was not required either for the expression of the *SL1028*, *SL3812* and *SL4433* genes in the growth conditions tested (data not shown), indicating that other regulators could link the expression of these genes with SPI-1. Additionally, we show that HilD can induce the expression of *gtgE-cat*, *phoH-cat*, *sinR-cat*, *lpxR-cat*, *SL3812-cat* and *SL4247-cat,* but not *SL1896-cat*, transcriptional fusions, in the *E. coli* MC4100 strain, and thus in the absence of other *Salmonella*-specific regulators or FlhDC; consistently, HilD bound to the regulatory regions of *gtgE*, *phoH*, *sinR*, *lpxR* and *SL4247,* but not to that of *SL1896*. Previously, by using chromatin immunoprecipitation-sequencing (ChIP-seq) and ChIP-qPCR, it was found that HilD binds *in vivo* to DNA regions associated with the *sinR*, *lpxR* and *SL4247 (STM14_5184*) *S*. Typhimurium 14028s genes; furthermore, it was shown that HilD can induce the expression of a *lpxR-lacZ* translational fusion in the *E. coli* AMD054 strain (*flhDC*^+^)^31^. Thus, HilD positively and directly regulates the expression of the *gtgE*, *phoH*, *sinR*, *lpxR* and *SL4247* genes, and positively but indirectly controls the expression of the *SL1896* gene.

**Figure 5:**
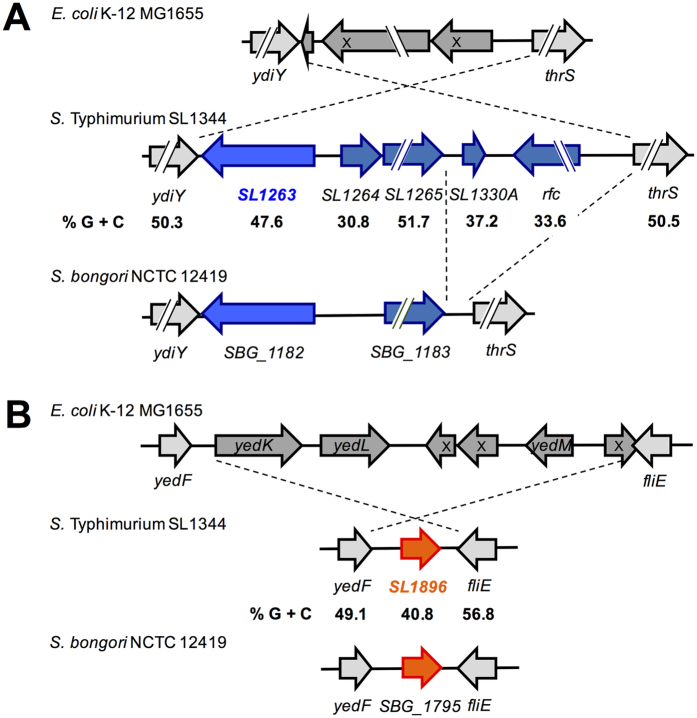
The *SL1263* and *SL1896* genes are located in *Salmonella* genomic islands that are absent in *E. coli* K-12. Schematic view of the regions between the *ydiY* and *thrS* ancestral genes (**A**), and the *yedF* and *fliE* ancestral genes (**B**), in *E. coli* K-12 MG1655, *S*. Typhimurium SL1344 and *S. bongori* NCTC 12419. The regions different in *S*. Typhimurium SL1344 with respect to those of *E. coli* K-12 MG1655, as well as the region present in *S*. Typhimurium SL1344 but not in *S. bongori* NCTC 12419, are indicated (see text for description). Pseudogenes are marked with an X. Broken arrows represent long genes. The G + C content for each of the *S*. Typhimurium SL1344 genes is shown.

The *gtgE*, *phoH*, *sinR*, *lpxR*, *SL4247* and *SL1896* genes are located in different *S*. Typhimurium chromosomal regions (Table S3 in Supplementary File 1). The *phoH* gene has a G + C content (50.7%) similar to the average G + C content of the *Salmonella* genome (52%), indicating that this is an ancestral gene; whereas the *gtgE*, *sinR*, *lpxR*, *SL4247* and *SL1896* genes have low G + C contents (34.4%, 39.7%, 47.6%, 46.6% and 40.8%, respectively), supporting that these genes were acquired for *S*. Typhimurium by horizontal transfer. Consistently, the *phoH* gene is highly conserved in most of bacteria and some archaea (Supplementary File 3)^48^ and the *gtgE*, *sinR* and *SL4247 (STM4310*) genes are located in *S.* Typhimurium genomic islands^6^,^49^,^50^,^51^,^52^. Genome context and BLAST analyses revealed that the *lpxR* and *SL1896* genes are also located in *S*. Typhimurium genomic islands (Fig. 5). The *lpxR (SL1263*) gene is located in a *S*. Typhimurium region that is absent in *E. coli* K-12, which is flanked by the *ydiY* and *thrS* genes, encoding a conserved putative protein and the enzyme threonyl-tRNA synthetase, respectively. This region also carries the *SL1264*, *SL1265*, *SL1330A* and *rfc (SL1266*) genes, which have low G + C content, with the exception of *SL1265*; thus, we denominated this region as island SL1263-66 (Fig. 5A). *SL1264* and *SL1330A* are genes of unknown function, whereas the *SL1265* and *rfc* genes are predicted to code for a DNA/RNA non-specific endonuclease and an O-antigen polymerase, respectively. In *E. coli* K-12, instead the SL1263-66 island, the *ydiY* and *thrS* genes flank the *arpB_1* and *arpB-2* pseudogenes (Fig. 5A). Interestingly, *S. bongori* contains the region spanning the *SL1263* and *SL1265* genes, but not the *SL1330A* and *rfc* genes, suggesting that *S.* Typhimurium acquired the SL1263-66 island by distinct horizontal transfer events; in agreement, the genes within this island show different G + C contents (Fig. 5A). On the other hand, the *SL1896* gene is present in *S*. Typhimurium and *S. bongori*, but not in *E. coli* K-12; it is located between the *yedF* and *fliE* genes, encoding a conserved putative protein and the flagellar basal-body protein FliE, respectively (Fig. 5B). In *E. coli* K-12, the *yedF* and *fliE* genes flank a region carrying the *yedK*, *yedL* and *yedM* genes, as well as the *yedN_1*, *yedN_2* and *intG* pseudogenes (Fig. 5B).

The products of the *gtgE*, *sinR*, *lpxR*, *SL4247* and *SL1896* genes are highly conserved in *S. enterica*, and in some cases also in *S. bongori*; furthermore, orthologs for SinR, LpxR, SL4247 and SL1896 are present in some other bacteria (Supplementary File S3).

A role in *Salmonella* virulence has been previously determined for *gtgE*, *lpxR* and *sinR*. The *gtgE* gene, located in the Gifsy-2 bacteriophage^49^, encodes a T3SS effector protein (GtgE) that is translocated into host cells, where it cleaves the Rab29, Rab-32 and Rab-38 GTPases; depletion of Rab-32 prevents activation of a pathway for *Salmonella* killing inside macrophages^53^,^54^,^55^. GtgE is present in *S*. Typhimurium but not in *S*. Typhi (Supplementary File 3); interestingly, the ectopic expression of GtgE allows *S*. Typhi to survive and replicate within macrophages and tissues from mice, a nonpermissive host^54^. Consistently, GtgE is required for the systemic disease caused by *S*. Typhimurium in mice^49^,^55^,^56^. GtgE can be secreted through both the T3SS-1 and the T3SS encoded in SPI-2 (T3SS-2)^53^,^56^; furthermore, its expression is induced in growth conditions favoring the expression of SPI-1 and also in those for SPI-2^56^,^57^. Therefore, the expression of *gtgE* is controlled by HilD in SPI-1-inducing conditions and probably by another regulator in SPI-2-inducing conditions, which would coordinate the secretion of GtgE through the T3SS-1 and T3SS-2, respectively. Similarly, the expression of *slrP*, also encoding an effector protein secreted through both T3SS-1 and T3SS-2, is controlled by HilD in SPI-1-inducing conditions and by the response regulator PhoP in SPI-2-inducing conditions^32^. In addition to *gtgE*, the Gifsy-2 bacteriophage carries other *Salmonella* virulence genes, such as *sodCI* and *sseI*^49^,^58^. The *lpxR* gene, located in the 1263–66 island, encodes a Ca^2+^-dependent outer membrane enzyme (LpxR) that removes the 3´-acyloxyacyl residue of lipid A, the hydrophobic anchor of lipopolysaccharide (LPS)^59^. LpxR is required for *S*. Typhimurium growth inside macrophages, probably by its activity on lipid A that could be beneficial to evade host immune surveillance, as well as by its negative effect on the amount of the inducible nitric oxide synthase, which would reduce the nitric oxide-mediated antibacterial cellular response^60^,^61^,^62^. In addition to HilD, the expression of *lpxR* in SPI-1-inducing conditions is positively regulated by SlyA, a MarR-like regulator^63^. HilD and SlyA cooperate to directly control the expression of *ssrAB* in SPI-1-inducing conditions (our unpublished results), which could also apply for *lpxR*. The other genes located in the SL1263-66 island (Fig. 5A) remain uncharacterized. The *sinR* gene, located in SPI-6 (also know as *Salmonella enterica* centisome 7 genomic island)^51^, encodes a putative LysR-family transcriptional regulator^50^. No targets of SinR have been determined; however, a *S*. Typhimurium *sinR* insertion mutant is attenuated in replication within macrophages, which supports its regulatory role^64^. SPI-6 also carries other *Salmonella* virulence genes, such as *pagN*, *sfaCD*, *sciG*, *rhs1* and those encoding a type 6 secretion system^64^,^65^,^66^. Whether the regulation by HilD implies that the *gtgE*, *lpxR* and *sinR* genes also have a role in the *Salmonella* invasion of host cells and thus in the intestinal infection, as for most other genes regulated by HilD, needs to be investigated.

The *phoH* gene was firstly characterized in *E. coli* K-12, as encoding a protein (PhoH) that has an ATP-binding activity, and as positively controlled by the transcriptional regulator PhoB in response to phosphate limitation^67^. It was found that PhoH is homologous to the N-terminal ATPase domain of superfamily I helicases^68^ and that *phoH* is not an essential gene in *E. coli* K-12^69^; however, even when PhoH orthologs are present in most of bacteria and some archaea (Supplementary File 3), its function remains unknown. Moreover, to our knowledge, there are not previous studies involving any other regulator, in addition to the PhoR/B system, in the expression of *phoH*. It is tempting to speculate that the regulation by HilD recruited the PhoH activity as a factor that can contribute to the *Salmonella* pathogenesis.

The *SL4247* and *SL1896* genes encode hypothetical proteins (Table S3 in Supplementary File 1). Interestingly, the *SL4747 (STM4310*) gene is upstream of the putative *rtsA*-*rtsB*-*SL4249(STM4313*)-*SL4248(STM4312*) operon^52^, which is directly regulated by HilD^23^,^27^. The *rtsA* and *rtsB* genes code for transcriptional regulators involved in the expression of the SPI-1 and flagellar genes^23^,^70^. On the other hand, the *SL1896* gene is located in a region containing a large cluster of flagellar genes (data not shown). However, our results show that HilD does not control the expression of *SL1896* through FlhDC, the master regulator of the flagellar/chemotaxis genes, neither through the SPI-1 regulators HilA and InvF (Table 1). It is known that HilD can also control gene expression through HilC, SprB, RtsA and SsrA/B^4^,^14^, and possibly, as suggested by our results, through SinR and SlyA; thus, HilD could act on *SL1896* through any of these regulators.

Whether or not the *phoH*, *SL4247* and *SL1896* genes, or even the *SL1028*, *SL3812* and *SL4433* genes that are co-expressed with SPI-1 but not regulated by HilD, have a role in *Salmonella* virulence is a matter of our current investigation.

Several studies support the notion that HilD induces the expression of its target genes mainly by counteracting the repression exerted by the histone-like nucleoid structuring protein (H-NS) on the respective promoters^21^,^22^,^27^,^29^,^71^,^72^. Genome-wide transcriptional and/or binding analyses support that H-NS represses *gtgE*, *sinR*, *lpxR*, *SL1896* and *SL4247*^73^,^74^,^75^; however, whether HilD induces the expression of these genes by acting as an anti-repressor of H-NS, and how it positively controls the expression of *phoH*, remains to be determined.

The method and parameters that we initially used in this study for clustering the *S*. Typhimurium SL1344 global gene expression results from the COLOMBOS database, were successful to identify *gtgE*, *phoH*, *sinR*, *lpxR*, *SL1896* and *SL4247,* as novel genes regulated by HilD. By using less-stringent clustering parameters, we found 34 additional genes whose pattern of expression can be linked to that of the SPI-1 genes (Table S4 in Supplementary File 1). Interestingly, between these genes are *SL1265* and *SL4248*, located in the *S*. Typhimurium genomic islands carrying *lpxR (SL1263*) and *SL4247*, respectively, as well as *slyA* that is positively regulated by HilD (our unpublished results). This strongly supports that more targets of HilD can be found among these 34 genes.

Our findings further expand the virulence regulon of HilD and reveal novel factors possibly involved in the pathogenesis of *Salmonella*.

## Methods

### Bioinformatics analyses

The *S*. Typhimurium SL1344 compendium in the COLOMBOS database (www.colombos.net) contains transcriptional expression values for 4655 genes, from 213 condition contrasts. We used this dataset and a clustering method to find genes co-expressed with SPI-1. Firstly, the *k*-means algorithm^76^ was applied 100 times to generate clusters of genes by their expression profiles, using K values of 1024 and 466. Then, consensuses of these clusters were obtained with the consensus clustering method^77^, using grouping frequencies of 40% and 60%, for the K values of 1024 and 466, respectively, to assign a gene in a consensus cluster. Finally, consensus clusters containing a particular gene, used as bait, were selected and the frequency with which a determined gene is present in these consensus clusters was obtained. Specifically, SPI-1 genes were used as the bait; then, the genes present in at least one selected consensus cluster were considered as genes co-expressed with SPI-1. The same procedure was followed when the novel genes found to be co-expressed with SPI-1 were used as the bait. For less-stringent clustering conditions, grouping frequencies of 30% and 60%, and of 40% and 50%, for the K values of 1024 and 466, respectively, were used to assign a gene in a consensus cluster. This clustering method with similar parameters has been successful to group genes of *Escherichia coli* K-12 with a related biological function (Sánchez, M., unpublished data).

Position-specific scoring matrices (PSSMs) 1 and 2, representing HilD-binding consensus sequences, were generated by using the consensus program^78^ and the HilD-binding sites reported by Oleknovich and Kadner and by Singer *et al.*, respectively^27^,^30^. Scanning of the regulatory regions of tested genes with these PSSMs was performed with the matrix-scan program^78^ using a *P* value of 1e-3. The resulting hits are reported with a corresponding significance score, which is a log-transformation of the *E*-value.

The orthologs for the products of the *gtgE*, *phoH*, *sinR*, *lpxR, SL1028*, *SL1896*, *SL3812*, *SL4247* and *SL4433* genes were downloaded from a database of orthologs detected as Reciprocal Best Hits using soft masking and Smith-Waterman alignments (http://popolvuh.wlu.ca/Orthologs)^79^.

### Media and culture conditions

Bacterial cultures were grown at 37 °C in LB medium containing 1% tryptone, 0.5% yeast agar and 1% NaCl, pH 7.5. When necessary, media were supplemented with ampicillin (200 μg ml^−1^), streptomycin (100 μg ml^−1^) or kanamycin (30 μg ml^−1^). Cultures for CAT assays were performed as described previously^14^,^42^.

### Construction of the mutant strain lacking *flhDC*

Bacterial strains used in this work are listed in Table S5 in Supplementary File 2. Non-polar deletion of the *flhDC* operon in the *S*. Typhimurium SL1344 strain was generated by the λRed recombinase system, as reported previously^80^, using the respective primers described in Table S6 in Supplementary File 2, generating the strain DTM90. This mutant strain was verified by PCR amplification and sequencing.

### Construction of plasmids

Plasmids and primers used in this work are listed in Tables S5 and S6, respectively, in Supplementary File 2. To construct the plasmids containing the transcriptional fusions *gtgE-cat, phoH-cat*, *sinR-cat*, *trg-cat*, *SL1028-cat, lpxR-cat, SL1896-cat*, *SL3812-cat, SL4247-cat* and *SL4433-cat*, DNA fragments containing the intergenic region upstream of *gtgE*, *phoH*, *sinR*, *trg*, *SL1028*, *lpxR*, *SL1896*, *SL3812*, *SL4247* or *SL4433* were amplified by PCR with the primer pairs gtgE-Rv1/gtgE-Fw2, phoH-Rv/phoH-Fw, sinRH3-Rv33/sinRB2-Fw44, trg-Rv1/trg-Fw2, SL1028-Rv1/SL1028-Fw2, SL1263-Rv55/SL1263-Fw77, SL1896-Rv1/SL1896-Fw2, SL3812-Rv1/SL3812-Fw2, SL4247-Rv1/SL4247-Fw2 and SL4433-Rv1/SL4433-Fw2, respectively. The PCR products were digested with BamHI and HindIII restriction enzymes and then cloned into the BamHI and HindIII sites of the vector pKK232-8, which carries a promotorless *cat* gene (Amersham Pharmacia LKB Biotechnology), generating plasmids pgtgE-cat, pphoH-cat, psinR-cat, ptrg-cat, pSL1028-cat, plpxR-cat, pSL1896-cat, pSL3812-cat, pSL4247-cat and pSL4433-cat. To construct the plasmid pK6-HilD, the *hilD* structural gene was amplified by PCR using the primer pair HilDK6-F/HilDexR-PstI and chromosomal DNA from WT *S*. Typhimurium SL1344 as template. This PCR product was digested with NcoI and PstI restriction enzymes and then cloned into the vector pMPM-K6Ω^81^ digested with the same restriction enzymes. pK6-HilD expresses HilD from an arabinose-inducible promoter.

### CAT assays

The chloramphenicol acetyl transferase (CAT) assays and protein quantification to calculate CAT specific activities were performed as previously described^82^.

### Statistical analysis

Results from CAT assays were analyzed using One-Way analysis of variance (ANOVA) with the Dunnett multiple comparison test for Table 1, or unpaired two-tailed Student’s *t* test for Figs 2 and 3. This statistical analysis was performed using Prism 5 program version 5.04 (GraphPad Software, San Diego, CA).

### Expression and purification of MBP-HilD

Maltose binding protein (MBP)-HilD was expressed in *E. coli* BL21/DE3 containing pMAL-HilD1 and purified by using an amylose column, as described previously^14^.

### EMSAs

DNA fragments containing the intergenic region upstream of *gtgE*, *phoH*, *sinR*, *lpxR*, *SL1896 and SL4247* were obtained by PCR amplification with the same primer pairs used to construct the respective transcriptional fusion to the *cat* reporter gene. DNA fragments containing the intergenic region upstream of *hilA,* used as a positive control, and *sigD* or *ppk*, used as internal negative controls, were obtained by PCR amplification with the primer pairs hilA2R-HindIII/hilA1F-BamHI, sigD-H3R/sigD-BHIF and PPK-Rv1/PPK-Fw1, respectively. PCR products were purified using the QIAquick PCR purification kit (Qiagen). Each PCR product (≈100 ng) was mixed with an equal amount of the PCR product of *sigD* or *ppk* and increasing concentrations of purified MBP-HilD in a binding buffer containing 10 mM Tris (pH 8.0), 50 mM KCl, 1 mM dithiothreitol (DTT), 0.5 mM EDTA, 5% glycerol and 10 μg ml^−1^ bovine serum albumin (BSA), in a final volume of 20 μl. Protein-DNA binding reactions were incubated at room temperature for 20 min and then separated by electrophoresis in 5% non-denaturing acrylamide gels in 0.5 X Tris-borate-EDTA buffer at room temperature. The DNA fragments were stained with ethidium bromide and visualized with an Alpha-Imager UV transilluminator (Alpha Innotech Corp.).

## Additional Information

**How to cite this article**: Martínez-Flores, I. *et al.* *In silico* clustering of *Salmonella* global gene expression data reveals novel genes co-regulated with the SPI-1 virulence genes through HilD. *Sci. Rep.* **6**, 37858; doi: 10.1038/srep37858 (2016).

**Publisher's note:** Springer Nature remains neutral with regard to jurisdictional claims in published maps and institutional affiliations.

## Supplementary Material

Supplementary File 1

Supplementary File 2

Supplementary File 3

## Figures and Tables

**Table 1:** Effect of the SPI-1 regulators HilD, HilA and InvF on the expression of the novel genes found to be co-expressed with SPI-1.

Transcriptional fusions	CAT-specific activity in *S*. Typhimurium strains			
	WT	Δ*hilD*	Δ*hilA*	Δ*invF*
*gtgE-cat*	1103 ± 89	465 ± 87*	1046 ± 106	1002 ± 159
*phoH-cat*	415 ± 19	198 ± 23*	413 ± 31	422 ± 37
*sinR-cat*	498 ± 10	161 ± 29*	455 ± 49	483 ± 37
*lpxR-cat*	2049 ± 289	7 ± 0.57*	2043 ± 217	2274 ± 67
*SL1896-cat*	72 ± 9	1 ± 2*	67 ± 7	79 ± 8
*SL4247-cat*	144 ± 16	7 ± 7*	156 ± 13	154 ± 11
*SL3812-cat*	268 ± 9	270 ± 21	273 ± 15	270 ± 11
*SL4433-cat*	316 ± 15	347 ± 28	301 ± 14	315 ± 13
*SL1028-cat*	31 ± 3	25 ± 7	29 ± 4	31 ± 1
*invF-cat*	2958 ± 157	18 ± 32*	43 ± 35	2926 ± 126

Expression of the *gtgE-cat*, *phoH-cat*, *sinR-cat*, *lpxR-cat*, *SL1896-cat, SL4247-cat, SL3812-cat, SL4433-cat, SL1028-cat* and *invF-cat* transcriptional fusions contained in plasmids pgtgE-cat, pphoH-cat, psinR-cat, plpxR-cat, pSL1896-cat, pSL4247-cat, pSL3812-cat, pSL4433-cat, pSL1028-cat and pinvF-cat, respectively, was tested in the WT *S*. Typhimurium strain SL1344 and its isogenic Δ*hilD*, Δ*hilA* and Δ*invF* mutants. CAT-specific activity was determined from samples collected of bacterial cultures grown for 9 h in LB medium at 37 °C. The data are the average of three independent experiments performed in duplicate. Standard deviations are indicated.

^*^Expression statistically different with respect to that shown by the same transcriptional fusions in the WT strain, *P*-value < 0.001.
