# Supplementary material for: In silico clustering of Salmonella global gene expression data reveals novel genes co-regulated with the SPI-1 virulence genes through HilD
Source: Sci Rep. 2016 Nov 25;6:37858. doi: 10.1038/srep37858 (PMC5122947; doi:10.1038/srep37858)
Supplement: Supplementary File 2 [file srep37858-s2.pdf]

Supplementary File 2

In silico **clustering of** ***Salmonella*** **global gene expression data reveals novel genes co-regulated with the SPI-1 virulence genes through HilD**

Irma Martínez-Flores, Deyanira Pérez-Morales, Mishael Sánchez-Pérez, Claudia C. Paredes, Julio Collado-Vides, Heladia Salgado and Víctor H. Bustamante

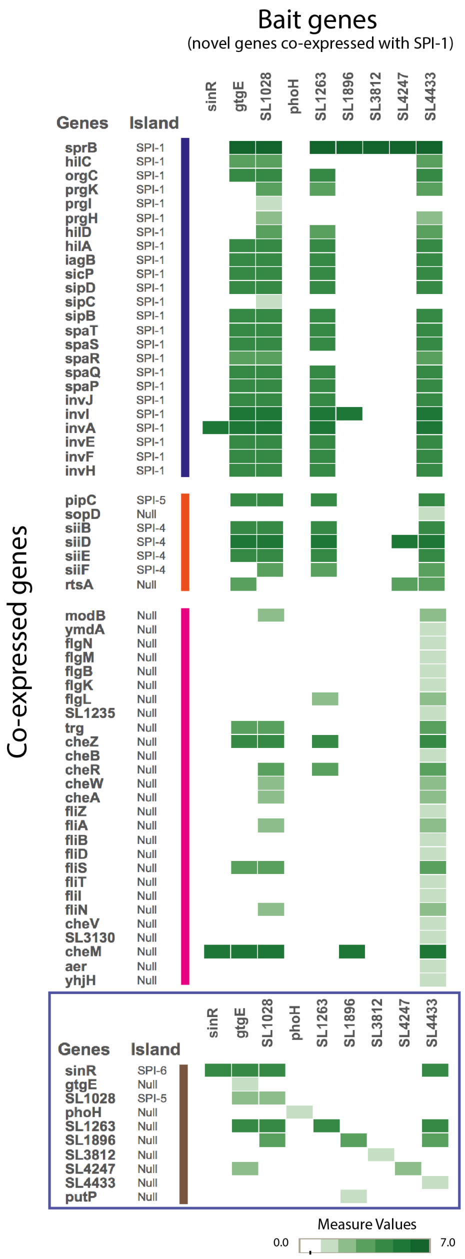

Figure S1. Clustering analysis using the genes found to be co-expressed with SPI-1 as the bait. The heat map represents the frequency with which each gene, shown at the left side of this Fig. (co-expressed genes), was clustered with the corresponding gene used as the bait, shown above the figure. The intensity of the green color in the heat map indicates the score obtained for each co-expressed gene, based in the color bar shown below the figure, ranging from Measure Values of 0 to 7. The scores for all co-expressed genes are displayed in Table S2 in Supplementary File 1. The co-expressed genes are classified in four groups: genes located in SPI-1, genes known to be co-regulated with SPI-1 that are located in other genomic islands, flagellar/chemotaxis genes and novel genes that are co-expressed with SPI-1, which are indicated with a dark blue, orange, pink and brown color bar, respectively. For a better visualization, the novel genes that are co-expressed with SPI-1 are boxed. The left side of this Fig. also show whether the co-expressed genes are located in any SPI. Null, indicates not located in any SPI.

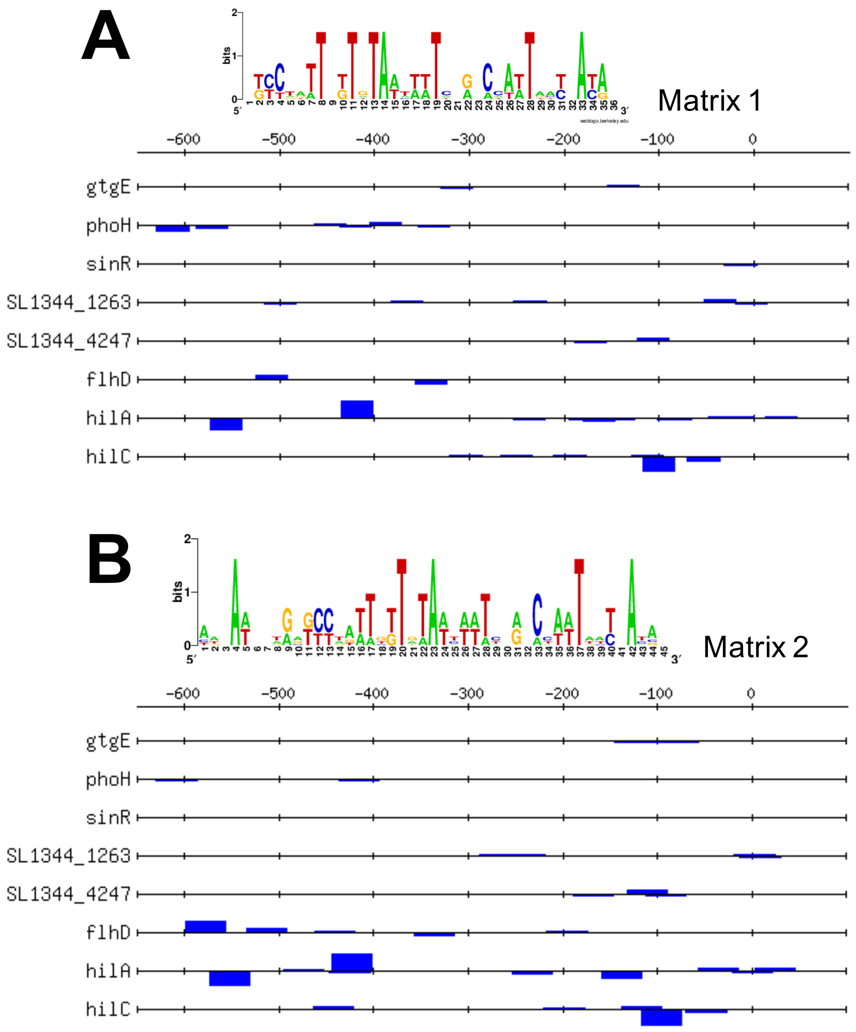

Figure S2. **Prediction of HilD-binding sites in the *gtgE*, *phoH*, *sinR*, *SL1263* and *SL4247* genes.** The region spanning 600 pb upstream and 100 bp downstream of each gene, with respect to the start codon, was scanned with position-specific scoring matrices (PSSMs) representing the HilD-binding consensus sequences reported by Oleknovich and Kadner (**A**) and Singer *et al*. (**B**)[1,2]. As positive controls, the regulatory regions of *flhD*, *hilA* and *hilC*, which are directly regulated by HilD, were also assessed. The sequence logo for each PSSM is shown. The predicted HilD-binding sites are displayed as blue boxes below or above thin lines representing the region of each gene, indicating localization in forward or reverse strands of DNA, respectively. The box height is proportional to the score obtained for each hit. By using matrix 1, known HilD-binding sites in *hilA* and *hilC* were detected with significance scores higher than 8.2, whereas predicted HilD-binding sites in *gtgE*, *phoH*, *sinR*, *SL1263* and *SL4247*, as well as in *flhD, hilA* and *hilC*, show significance scores lower than 4.7; the known HilD-binding site in *flhD* was not detected with this matrix. By using matrix 2, known HilD-binding sites in *flhD*, *hilA* and *hilC* were detected with significance scores higher than 8.0, whereas predicted HilD-binding sites in *gtgE*, *phoH*, *sinR*, *SL1263* and *SL4247*, as well as in *flhD, hilA* and *hilC*, show significance scores lower than 4.8. The significance index is a log-transformation of the *E*-value.

Strain or plasmid	Genotype or description	Source or reference
Strains		
S. Typhimurium		
SL1344	Wild type; *xyl*, *hisG*, *rpsL*; Sm^R	[3]
VV341	SL1344 Δ*hilA::kan-339*	[4]
JPTM4	SL1344 Δ*invF:kan*	[5]
JPTM25	SL1344 Δ*hilD*	[6]
DTM90	SL1344 Δ*flhDC::kan*	This study
E. coli		
MC4100	F$^-$ (*araD139*) Δ(*argF-lac*)*169* λ$^-$ e14$^-$ *flhD5301* Δ(*fruK-yeiR*)*725*(*fruA25*) *relA1 rpsL150*(Str^r) *rbsR22* Δ(*fimB-fimE*)*632*(::IS*1*) *deoC1*	[7]
DH10β	Laboratory strain	(Invitrogen)
Plasmids		
pKK232-8	pBR322 derivative containing a promotorless chloramphenicol acetyltransferase (*cat*) gene, Ap^R	[8]
pinvF-cat	pKK232-8 derivative containing a *invF-cat* transcriptional fusion from nucleotides -306 to +213[a]	[5]
philA-cat-410+446	pKK232-8 derivative containing a *hilA-cat* transcriptional fusion from nucleotides -410 to +446[a]	[5]
pssaG-cat	pKK232-8 derivative containing a *ssaG-cat* transcriptional fusion from nucleotides -232 to +361[a]	[5]
pgtgE-cat	pKK232-8 derivative containing a *gtgE-cat* transcriptional fusion from nucleotides -324 to +157[a]	This study
pphoH-cat	pKK232-8 derivative containing a *phoH-cat* transcriptional fusion from nucleotides -653 to +416[a]	This study
psinR-cat	pKK232-8 derivative containing a *sinR-cat* transcriptional fusion from nucleotides -715 to +157[a]	This study
ptrg-cat	pKK232-8 derivative containing a *trg-cat* transcriptional fusion from nucleotides -273 to +142[a]	This study
pSL1028-cat	pKK232-8 derivative containing a *SL1028-cat* transcriptional fusion from nucleotides -289 to +145[b]	This study
plpxR-cat	pKK232-8 derivative containing a *lpxR-cat* transcriptional fusion from nucleotides -162 to +287[b]	This study
pSL1896-cat	pKK232-8 derivative containing a *SL1896-cat* transcriptional fusion	

	from nucleotides -192 to +246[a]	This study
pSL3812-cat	pKK232-8 derivative containing a *SL3812-cat* transcriptional fusion from nucleotides -219 to +336[a]	This study
pSL4247-cat	pKK232-8 derivative containing a *SL4247-cat* transcriptional fusion from nucleotides -383 to +143[a]	This study
pSL4483-cat	pKK232-8 derivative containing a *SL4483-cat* transcriptional fusion from nucleotides -637 to +120[b]	This study
pKD46	pINT-ts derivative containing red recombinase system under an arabinose-inducible promoter, Ap^R	9
pKD4	pANTsγ derivative template plasmid containing the kanamycin cassette for λRed recombination, Ap^R	9
pMAL-HilD1	pMAL-c2X derivative expressing MBP-HilD from a *lac* promoter, Ap^R	5
pMPM-K6Ω	p15A derivative low-copy-number cloning vector, *ara* promoter, Kan^R	10
pK6-HilD	pMPM-K6Ω derivative expressing HilD from the arabinose-inducible promoter, Kan^R	This study

Table S5. Bacterial strains and plasmids used in this study. The coordinates for the *cat* fusions are indicated with respect to the primary transcriptional start site (a), described previously[11,12], or to the first base of the start codon (b), for each gene. Ap^R, ampicillin resistance; Sm^R, streptomycin resistance; Kan^R, kanamycin resistance.

Primer	Sequence (5'-3')	Target gene	RE
For *cat* transcriptional fusions and EMSAs			
gtgE-Rv1	GCAAAGCTTGTAGAGGGTGTTGCCTGTG	*gtgE*	HindIII
gtgE-Fw2	CCTGGATCCGAAGTGAGGGTGAAGCCG	*gtgE*	BamHI
phoH-Rv	CGAAAGCTTCCATGGATAGCACCTTGAGT	*phoH*	BamHI
phoH-Fw	CGAGGATCCAATATGGCTGGCTGGATCTG	*phoH*	BamHI
sinRH3-Rv33	GCCAAGCTTAGATTCCCTACTTCCGCCAC	*sinR*	HindIII
sinRB2-Fw44	ACCGGATCCAAAGTGCTGCATACCGAATCC	*sinR*	BamHI
trg-Rv1	AAAAAGCTTGGATGCCACCGAGAATGGAG	*trg*	HindIII
trg-Fw2	GCAGGATCCAGGGATGGTCGAAGATGC	*trg*	BamHI
SL1028-Rv1	CGCAAGCTTATTCTGGTATAAGTTGAAATAC<br>TG	*SL1028*	HindIII
SL1028-Fw2	GCAGGATCCATTGCTTCTTTCGTACCGG	*SL1028*	BamHI
SL1263-Rv55	TTGAAGCTTGTGGTCGTACTGAGTATGG	*SL1263*	HindIII
SL1263-Fw77	ATAGGATCCTGACAGCCGTTAGATATTC	*SL1263*	BamHI
SL1896-Rv1	CGAAAGCTTAATGGCGCTCGGATGTGCATT	*SL1896*	HindIII
SL1896-Fw2	CGAGGATCCCGACTGTCCGCAATCCATTA	*SL1896*	BamHI
SL3812-Rv1	CACAAGCTTCGACTTTGCCACCACACACG	*SL3812*	HindIII
SL3812-Fw2	TCTGGATCCGTGCGGAGTACCTTCAGC	*SL3812*	BamHI
SL4247-Rv1	CGAAAGCTTTCACGCCCAAGAGTACAACC	*SL4247*	HindIII
SL4247-Fw2	CGAGGATCCCATCATAATCGCGTCATCGC	*SL4247*	BamHI
SL4483-Rv1	CAGAAGCTTCTCTTTTGCATTCTCATCG	*SL4483*	HindIII
SL4483-Fw2	GATGGATCCAGGGCAAATAAGTGGCGTC	*SL4483*	BamHI
hilA2R-HindIII	GACAAGCTTTTCTGAGCGTAGCAGGG	*hilA*	HindIII
hilA1F-BamHI	ATCGGATCCCTCTGAGAACTATTTGC	*hilA*	BamHI
SigD-H3R	CGTTGTATAAGCTTTTTTGTAG	*sigD*	HindIII
SigD-BHIF	TCCCGACAGGATCCTTTTACCCATG	*sigD*	BamHI
PPK-Rv1	ATCGGATCCTCTGATTCCGAACAGCGTG	*ppk*	BamHI
PPK-Fw1	GCGAAGCTTAAATGCTAACCAGCTCAGTTC	*ppk*	HindIII
For *hilD* cloning			
HilDK6-F	GATACCATGGAAAATGTAACCTTTGTAAGTA<br>ATAG	*hilD*	NcoI
HilDexR-PstI	TCCCTGCAGAACAATGATATTGAATAGC	*hilD*	PstI
For *flhDC* deletion			
SflhDCH1P1	ATGGGAACAATGCATACATCCGAGTTGCTA<br>AAACACATTTAT*TGTAGGCTGGAGCTGCTTCG*	*flhDC*	
SflhDCH2P2	TTAAACAGCCTGTTCGATCTGTTCATCCAGC<br>AGTTGTGGAAT*CATATGAATATCCTCCTTAG*	*flhDC*	

Table S6. Primers used in this study. RE, restriction enzyme for which a site was generated in the primer. Underlined letters indicate the respective restriction-enzyme site in the primer. The sequence of the *flhDC* primers corresponding to the template plasmid pKD4 is in italic letters.

References

1. Singer, H. M., Kuhne, C., Deditius, J. A., Hughes, K. T. & Erhardt, M. The *Salmonella* Spi1 virulence regulatory protein HilD directly activates transcription of the flagellar master operon *flhDC*. *J Bacteriol* **196**, 1448-1457, doi:10.1128/JB.01438-13 (2014).
2. Olekhnovich, I. N. & Kadner, R. J. Role of nucleoid-associated proteins Hha and H-NS in expression of *Salmonella enterica* activators HilD, HilC, and RtsA required for cell invasion. *J Bacteriol* **189**, 6882-6890 (2007).
3. Hoiseth, S. K. & Stocker, B. A. Aromatic-dependent *Salmonella typhimurium* are non-virulent and effective as live vaccines. *Nature* **291**, 238-239 (1981).
4. Bajaj, V., Lucas, R. L., Hwang, C. & Lee, C. A. Co-ordinate regulation of *Salmonella typhimurium* invasion genes by environmental and regulatory factors is mediated by control of *hilA* expression. *Mol Microbiol* **22**, 703-714 (1996).
5. Bustamante, V. H. *et al.* HilD-mediated transcriptional cross-talk between SPI-1 and SPI-2. *Proc Natl Acad Sci U S A* **105**, 14591-14596 (2008).
6. Martínez, L. C. *et al.* Integration of a complex regulatory cascade involving the SirA/BarA and Csr global regulatory systems that controls expression of the *Salmonella* SPI-1 and SPI-2 virulence regulons through HilD. *Mol Microbiol* **80**, 1637-1656, doi:10.1111/j.1365-2958.2011.07674.x (2011).
7. Ferenci, T. *et al.* Genomic sequencing reveals regulatory mutations and recombinational events in the widely used MC4100 lineage of *Escherichia coli* K-12. *J Bacteriol* **191**, 4025-4029, doi:10.1128/JB.00118-09 (2009).
8. Brosius, J. Plasmid vectors for the selection of promoters. *Gene* **27**, 151-160 (1984).
9. Datsenko, K. A. & Wanner, B. L. One-step inactivation of chromosomal genes in *Escherichia coli* K-12 using PCR products. *Proc Natl Acad Sci U S A* **97**, 6640-6645 (2000).
10. Mayer, M. P. A new set of useful cloning and expression vectors derived from pBlueScript. *Gene* **163**, 41-46 (1995).
11. Kroger, C. *et al.* The transcriptional landscape and small RNAs of *Salmonella enterica* serovar Typhimurium. *Proc Natl Acad Sci U S A* **109**, E1277-1286, doi:10.1073/pnas.1201061109 (2012).
12. Ramachandran, V. K., Shearer, N. & Thompson, A. The primary transcriptome of *Salmonella enterica* Serovar Typhimurium and its dependence on ppGpp during late stationary phase. *PLoS One* **9**, e92690, doi:10.1371/journal.pone.0092690 (2014).
